# Ridge Polynomial Neural Network with Error Feedback for Time Series Forecasting

**DOI:** 10.1371/journal.pone.0167248

**Published:** 2016-12-13

**Authors:** Waddah Waheeb, Rozaida Ghazali, Tutut Herawan

**Affiliations:** 1 Faculty of Computer Science and Information Technology, Universiti Tun Hussein Onn Malaysia, Batu Pahat, Johor, Malaysia; 2 Computer Science Department, Hodeidah University, Hodeidah, Yemen; 3 Department of Information Systems, University of Malaya, Pantai Valley, Kuala Lumpur, Malaysia; 4 Department of Computer Science, Universitas Teknologi Yogyakarta, Yogyakarta, Indonesia; 5 AMCS Research Center, Yogyakarta, Indonesia; Jiangnan University, CHINA

## Abstract

Time series forecasting has gained much attention due to its many practical applications. Higher-order neural network with recurrent feedback is a powerful technique that has been used successfully for time series forecasting. It maintains fast learning and the ability to learn the dynamics of the time series over time. Network output feedback is the most common recurrent feedback for many recurrent neural network models. However, not much attention has been paid to the use of network error feedback instead of network output feedback. In this study, we propose a novel model, called Ridge Polynomial Neural Network with Error Feedback (RPNN-EF) that incorporates higher order terms, recurrence and error feedback. To evaluate the performance of RPNN-EF, we used four univariate time series with different forecasting horizons, namely star brightness, monthly smoothed sunspot numbers, daily Euro/Dollar exchange rate, and Mackey-Glass time-delay differential equation. We compared the forecasting performance of RPNN-EF with the ordinary Ridge Polynomial Neural Network (RPNN) and the Dynamic Ridge Polynomial Neural Network (DRPNN). Simulation results showed an average 23.34% improvement in Root Mean Square Error (RMSE) with respect to RPNN and an average 10.74% improvement with respect to DRPNN. That means that using network errors during training helps enhance the overall forecasting performance for the network.

## Introduction

Time series is a sequence of observations for a variable of interest made over time. Time series is used in many disciplines for things such as hourly air temperature, daily stock prices, weekly interest rates, monthly sales, quarterly unemployment rate, annual deaths from homicides and suicides, and electrocardiograph measurements. Time series can be categorized into different categories such as continuous and discrete time series, linear and nonlinear time series, and univariate and multivariate time series categories [1].

Univariate time series are obtained by recording a single phenomenon over time. Multivariate time series are recorded for more than one phenomenon over time [1]. A recording of a single phenomenon like annual volcano Carbon Dioxide (*CO*_2_) emissions is an univariate time series, while a (*CO*_2_) concentration of a gas furnace using (*CO*_2_) concentrations and input gas flow rate is an example of a multivariate time series.

One of the main objectives of analysing time series is forecasting [2]. Forecasting is needed in many areas, including, marketing, production planning, financial risk management, and crisis management. Forecasting is needed because it guides decisions, decreases dependence on chance, and makes dealing with the environment more scientific [3, 4].

Time series forecasting is defined as an estimation of the future behaviour of a time series using current and past observations [1]. Time series forecasting finds the relationship between past, present and future observations. Mathematically, univariate time series forecasting takes a series of data such as *x*_1_, *x*_2_, …, *x*_*N*_ to estimate future values such as *x*_*N*+*h*_, where the integer *h* is the forecast horizon. The forecast horizon is defined as the time period in the future to which forecasts are calculated.

Various methods for time series forecasting have been developed. From statistics-based to intelligence-based, there are a range of methods available to make a forecast. Conventional statistical methods such as Auto Regressive (AR), Auto Regressive Moving Average (ARMA) and exponential smoothing are linear-based methods that assume linear relationships between past values. The non-linear relationships found in most real time series data cannot be captured using these methods [5–8].

Intelligent methods such as Artificial Neural Networks (ANNs) have been successfully used in time series forecasting [6, 7, 9, 10]. ANN is an intelligence-based method which is inspired by biological nervous systems. During training, ANNs use historical data (i.e., current and past observations) to build a model that has the ability to forecast future observations.

ANNs have some advantages that attract researchers to use them in forecasting [7, 11]. First, they have a non-linear input-output mapping nature that allow ANNs to approximate any continuous function with an arbitrarily degree of accuracy. Second, the non-linear input-output mapping is generated with little priori knowledge about the non-linearity in the series, so ANNs are less susceptible to model misspecification than other parametric non-linear methods. Third, the generalization capabilities of ANNs in a non-stationary environment remain accurate and robust. Fourth, ANNs have the capability of tolerating the presence of chaotic components that are found in many time series.

ANNs can be grouped based on network structure into feedforward and recurrent networks [12]. In feedforward networks, the information moves in one direction only from the input nodes to the output nodes through one-way network connections (i.e., weights). On other hand, the connections between the units in recurrent networks can form a cycle.

One of the most used feedforward ANNs in time series forecasting is Multilayer Perceptrons (MLPs) [13]. Due to the multi-layered structure of MLPs, they need a large number of units to solve complex nonlinear mapping problems, which results in a low learning rate and poor generalization [14]. To overcome these drawbacks, different types of single layer higher order feedforward neural networks have been presented. Ridge Polynomial Neural Network (RPNN) [15] is a higher order feedforward neural network that maintains fast learning and powerful mapping properties which make it suitable for solving complex problems [13].

For time series forecasting, an explicit treatment for the dynamics involved is needed for neural network models because the behaviour of some time series signals are related to past inputs which present inputs depend on [16]. The explicit treatment of dynamics can be achieved using recurrent feedback. A recurrent version of the RPNN was proposed by [16]. This model is called the Dynamic Ridge Polynomial Neural Network (DRPNN). DRPNN uses the output value from the output layer as a feedback connection to the input layer. The idea behind recurrent network is to learn the network dynamics of the series over time. As a result, the trained network use its memory when forecasting [17]. RPNN and DRPNN have been successfully applied to forecast time series [9, 10, 13, 16] with DRPNN the most suitable for time series forecasting.

Instead of using network output feedback, network error feedback was used effectively with different models for time series forecasting as an additional input in the network [18–23]. Using network error as a feedback connection helps reduce the overall network error and increase forecasting accuracy. Due to the success of RPNNs, DRPNNs and models that use network error feedback for time series forecasting, in this study we propose the Ridge Polynomial Neural Network with Error Feedback (RPNN-EF). This paper is an extension of a report originally reported in the Second International Conference on Soft Computing and Data Mining (SCDM-2016) [24].

The contributions made by this study are as follows:

- We proposed Ridge Polynomial Neural Network with Error Feedback (RPNN-EF) for time series forecasting.
- The novelty of the proposed approach is that we incorporated the concepts of higher order terms, recurrence and error feedback in the proposed model.
- To overcome the stability and convergence problems that could occur due to existence of recurrent feedback in the RPNN-EF, a sufficient condition based on an approach that used adaptive learning rate was developed by introducing a Lyapunov function.
- A comparative analysis of the proposed model with RPNN and DRPNN was completed using four time series, star brightness, monthly smoothed sunspot numbers, daily Euro/Dollar exchange rate, and Mackey-Glass time-delay differential equation. The proposed model was compared with other models in the literature.

The remainder of this study is organized as follows. In Section 2, we review existing ridge polynomial neural network based models. In Section 3, we present the proposed model for time series forecasting. Section 4 describes the experimental design. Section 5 presents results and discussion. The conclusion and ideas for future works are given in Section 6.

## The Existing Ridge Polynomial Neural Network Based Models

Several feedforward neural networks have mapping capabilities for approximating reasonable functions [15]. A Multilayer perceptron (MLP) network is an example of these feedforward neural networks. MLP needs a large number of units to solve complex nonlinear mapping problems. Therefore, MLP is prone to a low learning rate and poor generalization [14]. To overcome multilayer network drawbacks, different types of single layer higher order feedforward neural networks were presented. One of these is the Pi-Sigma Neural Network (PSNN) which is a higher order feedforward neural network that consists of a single layer of trainable weights and product units in the output layer [25]. PSNN maintains powerful mapping capability and fast learning property without experiencing free parameter explosion problem [15]. PSNN demonstrated competent performance for various problems [25–27], however PSNN is not a universal approximator.

### Ridge Polynomial Neural Network (RPNN)

RPNN is a higher order feedforward neural network that was introduced to overcome the drawback of PSNN [15]. RPNN with arbitrary degree of accuracy can uniformly approximate any continuous function on a compact set in multidimensional input space [15]. Like PSNN, RPNN utilizes univariate polynomials which help to avoid the problem of free parameters explosion found in some types of higher order feedforward neural networks [15]. RPNN is constructed by adding different degrees of PSNNs during learning until a specified goal is achieved. It uses constructive learning, which is the growth of a small network structure during training until a specified goal is achieved.

**Fig 1:**
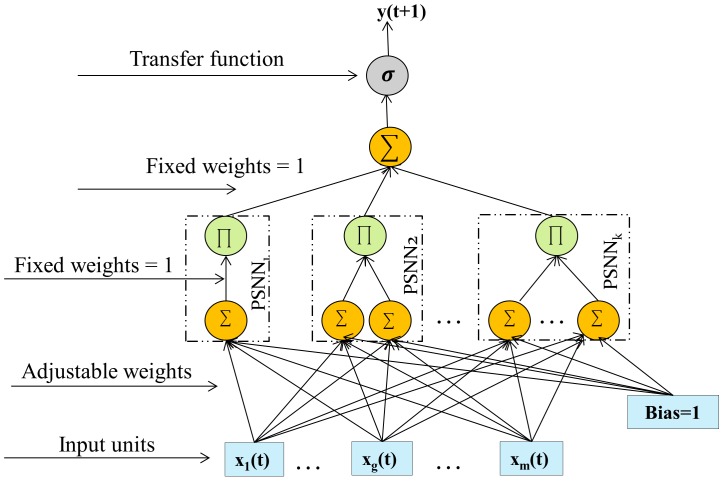
Ridge Polynomial Neural Network. PSNN stands for Pi-Sigma Neural Network.

Fig 1 shows a generic RPNN architecture. It can be seen that RPNN consists of only a single layer of adjustable weights which helps to speed the learning. The output of this network is as follows:

$$\begin{array}{c} y\left(t+1\right)\approx \sigma (\sum_{i=1}^{k}P_{i}\left(t+1\right)) \end{array}$$ (1a)

$$\begin{array}{c} P_{i}\left(t+1\right)=\prod_{j=1}^{i}(h_{j}\left(t+1\right)) \end{array}$$ (1b)

$$\begin{array}{c} h_{j}\left(t+1\right)=\sum_{g=1}^{m}w_{gj}x_{g}\left(t\right) \end{array}$$ (1c)

where *σ* is a non-linear activation function, *k* is the number of PSNN blocks, *m* is input vector dimension size, *w* is a trainable weight and *x* is the input.

RPNN has been used for different problems such as time series forecasting [13], function approximation [15], classification [15, 28] and pattern recognition [29]. It shows superior performance when compared to MLP.

### Dynamic Ridge Polynomial Neural Network (DRPNN)

DRPNN is the recurrent version of RPNN. It uses network output from the output layer as a feedback connection to the input layer. That means that DRPNN is provided with memories which retains information for later use [16]. According to [16], explicit treatment of dynamics is needed for neural network models due the fact that the behaviour of some time series is related to past values on which the present values depend upon. Therefore, DRPNN is more suitable than RPNN for time series forecasting as found in [9, 10, 16].

**Fig 2:**
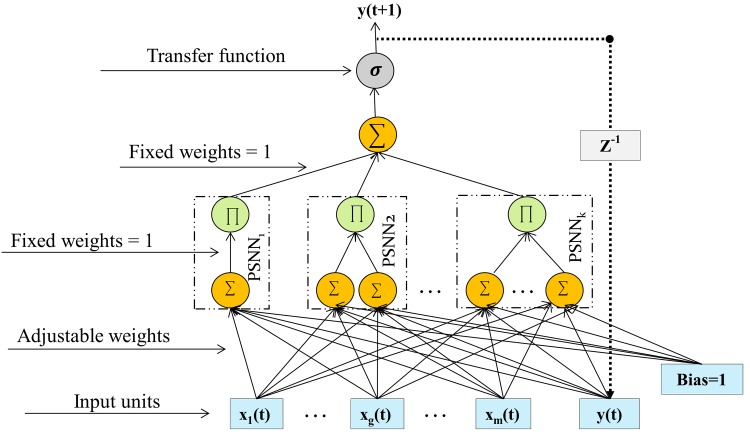
Dynamic Ridge Polynomial Neural Network. PSNN stands for Pi-Sigma Neural Network and *Z*^−1^ denotes the time delay operator.

The structure of DRPNN is shown in Fig 2. The difference between DRPNN and RPNN is the additional input node in DRPNN which is fed by the previous network output value. The output of DRPNN network is given by:

$$\begin{array}{c} y\left(t+1\right)\approx \sigma (\sum_{i=1}^{k}P_{i}\left(t+1\right)) \end{array}$$ (2a)

$$\begin{array}{c} P_{i}\left(t+1\right)=\prod_{j=1}^{i}(h_{j}\left(t+1\right)) \end{array}$$ (2b)

$$\begin{array}{c} h_{j}\left(t+1\right)=\sum_{g=1}^{m+1}w_{gj}Z_{g}\left(t\right) \end{array}$$ (2c)

$$\begin{array}{c} Z_{g}\left(t\right)=\{\begin{array}{cc} x_{g}\left(t\right) & 1\leq g\leq m\\ y(t) & g=m+1 \end{array} \end{array}$$ (2d)

where *k* is the number of PSNN blocks, *σ* is a non-linear transfer function, *m* is the input vector dimension size, *w* is a trainable weight, *x* is the input and *y*(*t*) is network output at a previous time step.

## The Proposed Ridge Polynomial Neural Network with Error Feedback Model

Feedforward and recurrent networks have been used for time series forecasting. Recurrent networks have an advantage over feedforward networks in time series forecasting [9, 16]. The reason for this is due to the behaviour of some time series in which present inputs depends on past inputs. Therefore, explicit treatment of dynamic is needed and is achieved using recurrence. By using recurrent feedback, the network takes advantage on external inputs as well as the entire history of the system inputs [9, 16]. Network output or network error are feedback connections that were used in the literature as an additional input into the network [9, 16, 18, 23]. Therefore, making an explicit treatment of dynamic.

### Feedback Error Learning

Learning from error is not a new concept for neural networks. Backpropagation algorithm (BP) is widely used to train neural networks and is based on the concept of learning from error. BP calculates the difference between the desired output and network output, which is called error, and uses this error to direct training. Error Minimized Extreme Learning Machine (EM-ELM) [30] takes advantage of errors to control the growth of the network. It adds random hidden nodes singly or in a group and incrementally updates the weights to minimize errors in the training set.

Another way to take advantage of errors is by using network error as a feedback connection. Different variations of network error have been given by different researchers. In [18, 19], the authors calculate error by taking the difference between network output at time *t* + 1 and the desired value at time *t*. They used this error with a state space Neural Network (ssNN) for short-term temperature forecasting and with MLP for forecasting hourly energy consumption in buildings. The presented results demonstrated the successful capture of the dynamical behaviour of the models.

Instead of using the difference between network output at time *t* + 1 and the desired value at time *t*, the authors in [20–23] used the difference between desired value and network output at time *t* + 1. In [20], the authors proposed another way to use network errors. In the beginning, they initialized the training of an MLP network using training samples to find the initial structure for MLP. Then, using the initialized network the absolute forecasting error was calculated for each training sample and stored as an additional input to each sample. This was followed by the addition of an additional layer with the same number of hidden nodes found in the first hidden layer. This new structure was trained using new training samples. This process continues until the testing set’s square error becomes less than the specified goal. The experiment proved that their method is better than a traditional MLP. However, adding more hidden layers to a network leads to a large number of free parameters, and results in longer training time and could cause poor generalization.

Another simple use of error was used in [21–23]. Error was calculated and inserted into Adaptive Neuro-Fuzzy Inference System (ANFIS) [21], Recurrent Nonlinear Autoregressive Moving Average (Rcurrent NARMA) [22] and Functional Neural Network (FNN) [23] in the next time step. The objective of using error as an input is to reduce the overall network error.

Adaptive control studies also take advantage of error learning which is called feedback error learning (FEL) [31–33]. FEL was proposed to establish a computational model of the cerebellum for learning motor control with interval models in the central nervous system [33].

Based on these studies, the main objective of using network error as an additional spatial dimension in the input space is to reduce the overall network error. That means showing a network the difference between the desired output and its output during training could enhance the overall forecasting performance for the network.

### Network Structure and Weights Learning

**Fig 3:**
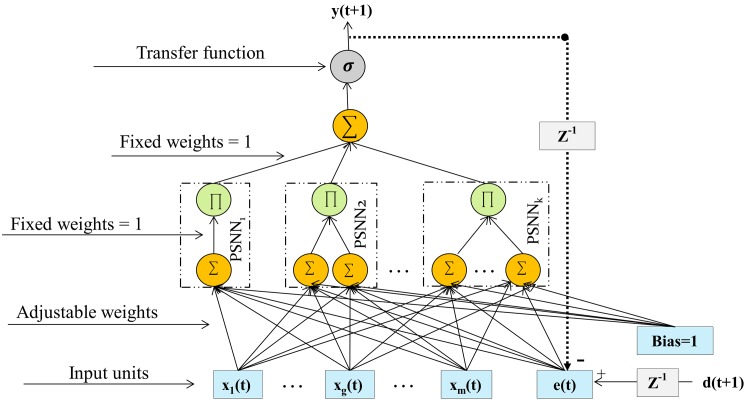
Ridge Polynomial Neural Network with Error Feedback. PSNN stands for Pi-Sigma Neural Network, *d*(*t* + 1) is the desired output at time *t* + 1 and *Z*^−1^ denotes the time delay operator.

RPNN-EF is constructed from a number of increasing order of Pi-Sigma units (PSNNs) with the addition of a recurrent connection from the output layer to the input layer. This recurrent connection is fed by network error, thus allowing the network to see errors in previous samples. Fig 3 shows a generic network architecture for RPNN-EF. It can be seen from Fig 3 that the only learnable connections are the connections that link the nodes in the input layer with the nodes of the first summing layer.

Assuming that *M* is the number of external inputs *x*(*t*) in the network, and that *e*(*t*) is the network error for RPNN-EF in a previous time step. The overall inputs to the network are the concatenation of *x*(*t*) and *e*(*t*), and are referred to as *Z*(*t*) as shown below:

$$\begin{array}{c} Z_{g}\left(t\right)=\{\begin{array}{cc} x_{g}\left(t\right) & 1\leq g\leq m\\ e(t)=d(t)-y(t) & g=m+1 \end{array} \end{array}$$ (3)

From Eq (3), network output at time *t* + 1, which is denoted by *y*(*t* + 1), is calculated as follows:

$$\begin{array}{c} y\left(t+1\right)\approx \sigma (\sum_{i=1}^{k-1}\hat{P_{i}\left(t+1\right)}+P_{k}\left(t+1\right)) \end{array}$$ (4a)

$$\begin{array}{c} P_{k}\left(t+1\right)=\prod_{j=1}^{k}(h_{j}\left(t+1\right)) \end{array}$$ (4b)

$$\begin{array}{c} h_{j}\left(t+1\right)=\sum_{g=1}^{m+1}w_{gj}Z_{g}\left(t\right) \end{array}$$ (4c)

where *k* is the number of Pi-Sigma blocks (PSNNs), *P*_*k*_(*t* + 1) is the output at time (*t* + 1) of last added PSNN block, $\hat{P_{i}\left(t+1\right)}$ is *P*_*i*_(*t* + 1) after its weights are frozen, *σ* is the sigmoid activation function, *h*_*j*_(*t* + 1) is the net sum of the sigma unit *j* in the last added PSNN block, and *w*_*gj*_ is the adjustable weights between inputs and sigma units.

Like RPNN and DRPNN, RPNN-EF uses a constructive learning algorithm based on the asynchronous updating rule of Pi-Sigma units. RPNN-EF adds a Pi-Sigma block of increasing order to its structure when the relative different between the current and the previous errors is less than a specified threshold value. RPNN-EF updates its weights using the Real Time Recurrent Learning algorithm [34]. A standard error measurement used for training the network is the sum squared error:

$$\begin{array}{c} E\left(t+1\right)=\frac{1}{2}\sum_{}^{}e{\left(t+1\right)}^{2} \end{array}$$ (5)

where

$$\begin{array}{c} e(t+1)=d(t+1)-y(t+1) \end{array}$$ (6)

where *d*(*t* + 1) is the desired output and *y*(*t* + 1) is the network output as shown in Eq (4a). At every time, the weights between inputs *g* and sigma *l* are updated as follows:

$$\begin{array}{c} \Delta w_{gl}=-\eta *(\frac{\partial E(t+1)}{\partial w_{gl}}) \end{array}$$ (7)

where *η* is the learning rate. The value of $\frac{\partial E(t+1)}{\partial w_{gl}}$ is determined as:

$$\begin{array}{c} \frac{\partial E(t+1)}{\partial w_{gl}}=\frac{\partial E(t+1)}{\partial e(t+1)}*\frac{\partial e(t+1)}{\partial w_{gl}} \end{array}$$ (8)

From Eq (5), we have:

$$\begin{array}{c} \frac{\partial E(t+1)}{\partial w_{gl}}=e\left(t+1\right)*\frac{\partial e(t+1)}{\partial w_{gl}} \end{array}$$ (9)

$$\begin{array}{c} \frac{\partial e(t+1)}{\partial w_{gl}}=\frac{\partial e(t+1)}{\partial y(t+1)}*\frac{\partial y(t+1)}{\partial P_{k}\left(t+1\right)}*\frac{\partial P_{k}\left(t+1\right)}{\partial w_{gl}} \end{array}$$ (10)

From Eqs (4) and (6), we have:

$$\begin{array}{c} \frac{\partial e(t+1)}{\partial w_{gl}}=-1*{\left(y(t+1)\right)}^{{}^{\prime }}*(\prod_{j=1,j\neq l}^{k}h_{j}\left(t+1\right))*(Z_{g}\left(t\right)+w_{(m+1)l}*\frac{\partial e(t)}{\partial w_{gl}}) \end{array}$$ (11)

Assuming *D*^*E*^ is the dynamic system variable, which is defined as a set of quantities that summarizes all the information about the past behaviour of the system that is needed to uniquely describe its future behaviour [12], *D*^*E*^ is:

$$\begin{array}{c} D_{gl}^{E}\left(t+1\right)=\frac{\partial e(t+1)}{\partial w_{gl}} \end{array}$$ (12)

Substituting Eqs (11) into (12), we have:

$$\begin{array}{c} D_{gl}^{E}\left(t+1\right)=\frac{\partial e(t+1)}{\partial w_{gl}}=-1*{\left(y(t+1)\right)}^{{}^{\prime }}*(\prod_{j=1,j\neq l}^{k}h_{j}\left(t+1\right))*(Z_{g}\left(t\right)+w_{(m+1)l}*D_{gl}^{E}\left(t\right)) \end{array}$$ (13)

For simplification, the initial values for $D_{gl}^{E}\left(t\right)=0$, and *e*(*t*) = 0.5 to avoid a zero value of $D_{gl}^{E}\left(t\right)=0$ [9, 16]. The weights updating rule is derived by substituting Eqs (9) and (13) into (7), as follows:

$$\begin{array}{c} \Delta w_{gl}=-\eta *e\left(t+1\right)*D_{gl}^{E}\left(t+1\right) \end{array}$$ (14)

Finally,

$$\begin{array}{c} w_{gl}^{new}=w_{gl}^{old}+\Delta w_{gl} \end{array}$$ (15)

### Stability issue

The ability to model the behaviour of arbitrary dynamical systems is one of the most useful properties of recurrent networks. The presence of a recurrent connection in RPNN-EF is expected to enhance its forecasting performance. Despite its potential of RPNN-EF feedback, the problems of complexity and difficult training could occur in RPNN-EF, as found in DRPNN [9]. These problems are summarized in two main points. First, calculating the gradients and updating the weights of a recurrent network is much more difficult than in a feedforward network due to dynamic system variables that affect both the gradient and the output. Second, learning could become unstable because the learning error may not monotonically decrease causing long convergence times.

In order to tackle these problems, a sufficient condition for the convergence of DRPNN was derived based on the stability theorem for a feedback network proposed by Atiya [35]. The aim of this theorem is to adjust the weights of the network to generate network outputs that are as close as possible to the desired output [9]. However, this solution could be too restrictive where a large network is necessary [35] or when working with constructive learning because it stops training with a small number of hidden units.

To overcome the stability and convergence problems of RPNN-EF, this study uses sufficient condition based on an approach that uses an adaptive learning rate developed by introducing a Lyapunov function.

First, let us define a Lyapunov function as follows:

$$\begin{array}{c} V\left(t\right)=\frac{1}{2}e^{2}\left(t\right) \end{array}$$ (16)

where *e*(*t*) represents error which is calculated by differencing the desired value from the predicted value. We use this error function because the RPNN-EF model is used to minimize it.

According to Eq (16), the change in the Lyapunov function is determined by:

$$\begin{array}{c} \Delta V\left(t\right)=V\left(t+1\right)-V\left(t\right)=\frac{1}{2}[e^{2}\left(t+1\right)-e^{2}\left(t\right)] \end{array}$$ (17)

The error difference can be represented by [36, 37]

$$\begin{array}{c} e(t+1)=e(t)+\Delta e(t) \end{array}$$ (18)

$$\begin{array}{c} e\left(t+1\right)≅e\left(t\right)+{\left[\frac{\partial e(t)}{\partial w}\right]}^{T}\Delta w \end{array}$$ (19)

where △*w* represents the weight change. Based on Eqs (13) and (14), we have:

$$\begin{array}{c} e\left(t+1\right)≅e\left(t\right)-\eta *e\left(t\right)*{\left[D^{E}\left(t\right)\right]}^{T}*D^{E}\left(t\right) \end{array}$$ (20)

$$\begin{array}{c} e\left(t+1\right)≅e\left(t\right)(1-\eta *{\left[D^{E}\left(t\right)\right]}^{T}*D^{E}\left(t\right)) \end{array}$$ (21)

From Eqs (17) and (21), △*V*(*t*) is represented as

$$\begin{array}{c} \Delta V\left(t\right)=\frac{1}{2}\eta *e^{2}\left(t\right)*{\left[D^{E}\left(t\right)\right]}^{T}*D^{E}\left(t\right)\left(\eta *{\left[D^{E}\left(t\right)\right]}^{T}*D^{E}\left(t\right)-2\right) \end{array}$$ (22)

$$\begin{array}{c} \Delta V\left(t\right)=\frac{1}{2}\eta *e^{2}\left(t\right)*{\left(∥D^{E}\left(t\right){∥}_{F}\right)}^{2}\left(\eta *{\left(∥D^{E}\left(t\right){∥}_{F}\right)}^{2}-2\right) \end{array}$$ (23)

where ∥.∥_*F*_ is the Frobenius norm which is calculated using a trace function [38].

A sufficient condition to ensure stability is △*V*(*t*) < 0. Therefore, Eq (23) leads to:

$$\begin{array}{c} 0<\eta <\frac{2}{{\left(∥D^{E}\left(t\right){∥}_{F}\right)}^{2}} \end{array}$$ (24)

Eq (24) suggests an upper bound of *η* for a sufficient condition to ensure stability in RPNN-EF.

### Constructive Learning Algorithm for the RPNN-EF

The proposed RPNN-EF is trained by the constructive learning algorithm based on the asynchronous update rule for PSNN. That means that the network structure grows from small to large as network learning proceeds until the desired level of specified error is reached. Before presenting the algorithm, we need the following notations-*ϵ*_*threshold*_: threshold Mean Squared Error (MSE) for the training phase; *ϵ*_*c*_, *ϵ*_*p*_: the training MSE’s for the current epoch and previous epoch, respectively; *r*: threshold for successive addition of a new PSNN blocks; *η*: initial learning rate; *δ*_*r*_, *δ*_*η*_: decreasing factors for *r* and *η*, respectively; *k*: degree of PSNN, as well as *Epoch*_*ID*_, and *Epoch*_*threshold*_: number of training epochs and maximum number of epochs to finish training, respectively.

The pseudo code used for RPNN-EF to update its weights is as follows:

**Algorithm 1** Constructive Leaming Algorithm for the RPNN-EF

Set *Epoch*_*ID*_ = 0.

Assign suitable values to *ϵ*_*threshold*_, *η*, *r*, *δ*_*r*_, *δ*_*η*_ and *Epoch*_*threshold*_.

**loop**

 Calculate *P*_*k*_ using Eq (4b)

A:

 **for all** training samples **do**

  Calculate actual network output using Eq (4a)

  Update weights by applying the asynchronous update rule in Eq (15)

  **if** *η* outside the bounds in Eq (24) **then**     ⊳Check stability issue

   Stop learning

  **end if**

 **end for**

 Calculate current epoch’s error (*ϵ*_*c*_)

 **if** *ϵ*_*c*_ < *ϵ*_*threshold*_ or *Epoch*_*ID*_ > *Epoch*_*threshold*_ **then**

  Stop learning

 **end if**

 *Epoch*_*ID*_ ← *Epoch*_*ID*_ + 1

 *ϵ*_*p*_ ← *ϵ*_*c*_

 **if**|(*ϵ*_*c*_ − *ϵ*_*p*_)/*ϵ*_*p*_|≥*r* **then**

  Go to Step A

 **else**

$\hat{P_{k}}\leftarrow P_{k}$

  *r* ← *r* ∗ *δ*_*r*_

  *η* ← *η* ∗ *δ*_*η*_

  *k* ← *k* + 1

 **end if**

**end loop**

## Experimental Design

This section provides a step by step methodology describing the design of a neural networks to forecast time series.

### Time Series used in the Experiments

Four time series were used in this study, namely star brightness (StarBrightness), monthly smoothed sunspot numbers (Sunspot), daily Euro/Dollar exchange rate (EUR/USD), and Mackey-Glass time-delay differential equation (Mackey-Glass).

Star brightness was recorded for 600 successive nights at midnight. This series was scaled by a factor of 1/30. This series was obtained from [39]. The monthly smoothed sunspot time series was downloaded from [40]. The sunspot time series was seen as a chaotic system with noise and is sensitive to initial conditions [41]. A sub-series in the sunspot time series from November 1834 to June 2001 consisting of 2, 000 months was selected. This interval was also selected by other researches [42, 43]. The third series is the daily Euro/Dollar (EUR/USD) exchange rate. The data set contains 781 observations covering the period from January 3, 2005 to December 31, 2007 [44]. The data was collected from [45, 46]. The last time series was generated from the Mackey–Glass time-delay differential equation which is defined as follows:

$$\begin{array}{c} \frac{dx}{dt}=\beta x\left(t\right)+\frac{\alpha x(t-\tau )}{1+x^{10}\left(t-\tau \right)} \end{array}$$ (25)

where *t* is a variable, *x* is a function of *t*, and *τ* is the time delay. The initial values of the series are *α* = 0.2, *β* = −0.1, *x*(0) = 1.2, and *τ* = 17. It is known that with this setting the series shows chaotic behaviour. From the generated time series, 1000 data points were extracted as explained in [47]. This series can be found in the file mgdata.dat in MATLAB [47] or in https://raw.githubusercontent.com/dodikk/neuro-mut/master/src/NetworkConverter/Samples/mgdata.dat.

**Fig 4:**
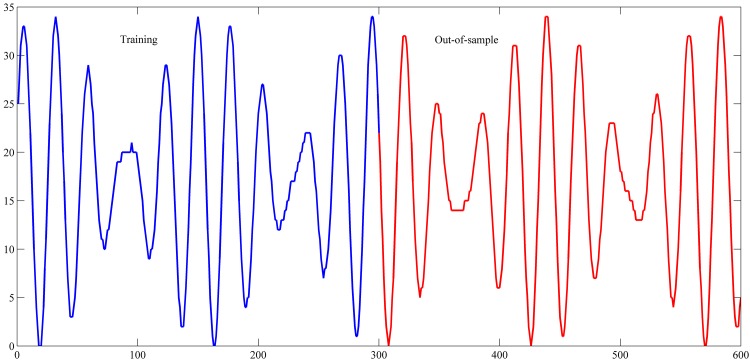
Star brightness time series.

**Fig 5:**
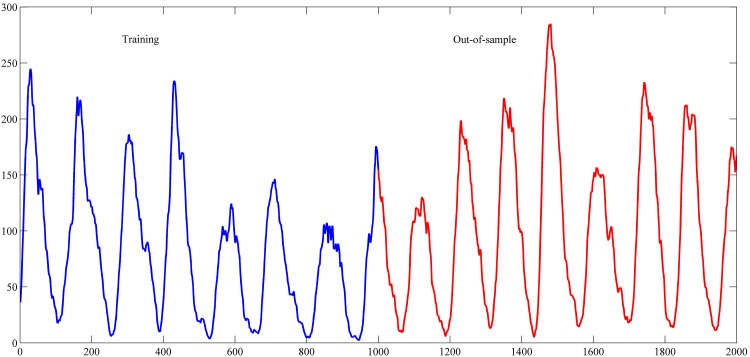
Monthly smoothed sunspot numbers time series from November 1834 to June 2001.

**Fig 6:**
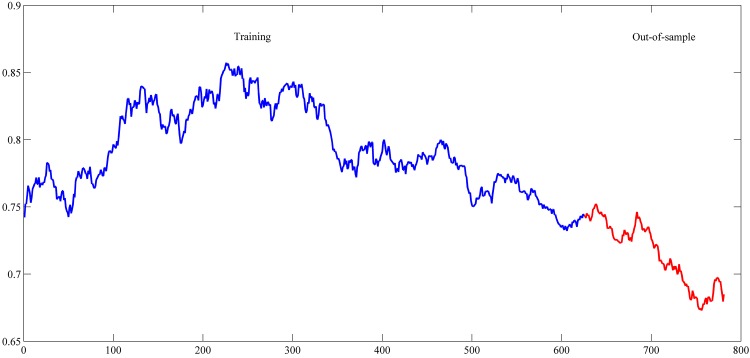
Daily Euro/Dollar exchange rate time series from January 3, 2005 to December 31, 2007.

**Fig 7:**
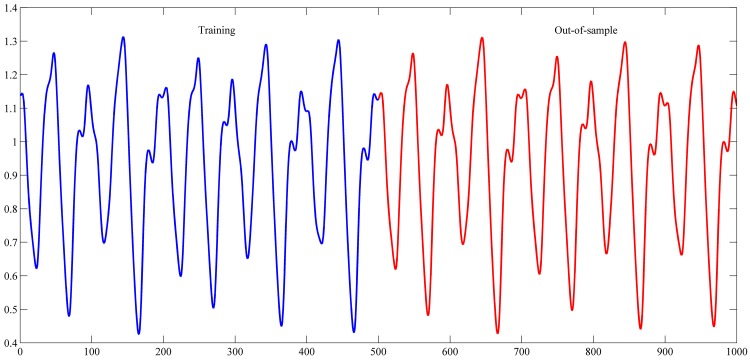
Mackey–Glass time-delay differential equation time series.

The settings used in this study for these series are shown in Table 1. These settings were also used in the studies of [42–44, 48–59]. The used intervals for training and out-of-sample sets are shown in Figs 4–7.

**Table 1:** Time series information.

	Input–output data pairs	Training samples#	Out-of-sample samples#
StarBrightness	[*x*(*t* − 2), *x*(*t* − 1), *x*(*t*); *x*(*t* + 1)]	300	300
Sunspot	[*x*(*t* − 4), *x*(*t* − 3), *x*(*t* − 2), *x*(*t* − 1), *x*(*t*); *x*(*t* + 1)]	1000	1000
EUR/USD	[*x*(*t* − 10), *x*(*t* − 5), *x*(*t*); *x*(*t* + 5)]	625	156
Mackey–Glass	[*x*(*t* − 18), *x*(*t* − 12), *x*(*t* − 6), *x*(*t*); *x*(*t* + 6)]	500	500

### Network Topology and Training

Network model topology describes the architecture of the network models and the way that the network is organized. The selected network topology was directly trained on the training set and tested on the out-of-sample set. Training was performed by repeatedly showing the network examples of inputs, paired with the desired output. During training, the difference between the desired and actual outputs was computed in order to update network weights.

Network topology and training parameters that were used in this study are shown in Table 2. Most of the settings are either based on previous works found in the literature [9, 10, 16] or by trial and error.

**Table 2:** Network topology and training.

Setting	Value
Number of input units	Input points which given in Table 1
Number of output units	One unit
Activation function	Sigmoid function
Number of Pi-Sigma block (PSNN)	The network’s order was incrementally grown from 1 to 5
Stopping criteria for RPNN	Maximum number of epochs = 3000 or, After accomplishing the 5th order network learning.
Stopping criteria for DRPNN & RPNN-EF	Maximum number of epochs = 3000 or, After accomplishing the 5th order network learning or, Network learning becomes unstable.
Initial weights range	[-0.5, 0.5]
Momentum range	[0.4-0.8]
Learning rate (*η*) range	[0.01-1]
Decreasing factors for n (*δ*_*η*_)	0.8
Threshold for successive addition of a new PSNN (*r*)	[0.00001, 0.1]
Decreasing factors for threshold (*δ*_*r*_)	[0.05, 0.2]

Since this study used the sigmoid transfer function, similar to [9, 16], the data was scaled in the range [0.2, 0.8]. This is to avoid getting network outputs too close to the two endpoints of the sigmoid function [10]. The equation to scale the data is given by:

$$\begin{array}{c} \dot{x}=\left(max_{new}-min_{new}\right)*(\frac{x-min_{old}}{max_{old}-min_{old}})+min_{new} \end{array}$$ (26)

where $\dot{x}$ refers to the normalized value, x refers to the observation value, *min*_*old*_, and *max*_*old*_, are the respective minimum and maximum values of all observations, respectively. *min*_*new*_, and *max*_*new*_, refer to the minimum and maximum of the new scaled series.

### Performance Metrics

In this study, network performance was evaluated using commonly used metrics for time series forecasting such as Root Mean Squared Error (RMSE), Normalized Mean Squared Error (NMSE), Mean Absolute Error (MAE) and Signal to Noise Ratio (SNR). This study carried out t-tests with a significance level of 0.05 to highlight significant performance. The equation for these metrics are given by:

Root Mean Squared Error (RMSE):

$$\begin{array}{c} RMSE=\sqrt{\frac{1}{N}\sum_{i=1}^{N}(y_{i}-{\hat{y_{i})}}^{2}} \end{array}$$ (27)

Normalized Mean Squared Error (NMSE):

$$\begin{array}{c} NMSE=\frac{1}{N\sigma^{2}}\sum_{i=1}^{N}(y_{i}-{\hat{y_{i})}}^{2} \end{array}$$ (28)

$$\begin{array}{c} \sigma^{2}=\frac{1}{N-1}\sum_{i=1}^{N}{\left(y_{i}-\bar{y}\right)}^{2} \end{array}$$ (29)

$$\begin{array}{c} \bar{y}=\frac{1}{N-1}\sum_{i=1}^{N}y_{i} \end{array}$$ (30)

Mean Absolute Error (MAE):

$$\begin{array}{c} MAE=\frac{1}{N}\sum_{i=1}^{N}|y_{i}-\hat{y_{i}}| \end{array}$$ (31)

Signal to Noise Ratio (SNR):

$$\begin{array}{c} SNR=10*\log_{10}\left(Sigma\right) \end{array}$$ (32)

$$\begin{array}{c} Sigma=\frac{m^{2}*N}{SSE} \end{array}$$ (33)

$$\begin{array}{c} m=max(y_{i}) \end{array}$$ (34)

$$\begin{array}{c} SSE=\sum_{i=1}^{N}(y_{i}-{\hat{y_{i})}}^{2} \end{array}$$ (35)

where *N*, *y* and $\hat{y}$ represent the number of out-of-sample data, actual output and network output, respectively.

## Results and Discussions

In this section, the simulation results for the forecasting of star brightness, monthly smoothed sunspot numbers, daily Euro/Dollar (EUR/USD) exchange rate, and Mackey-Glass time-delay differential equation are presented.

### Best Average Simulation Results

Since network training is significantly influenced by its initial internal state, which involves different initial learning parameters and different sets of random weights, an average of 30 independent simulations were performed for all neural networks in order to obtain fair and more robust comparative evaluations. The average performance for the various neural network architectures are shown in Tables 3–6. The results shown in these tables are the de-normalized results. That means, we de-normalized the forecasted value and compared it with the original desired value.

**Table 3:** Root Mean Squared Error (RMSE) improvement of RPNN-EF to RPNN and DRPNN.

	RPNN	DRPNN	RPNN-EF	Improvement to RPNN %	Improvement to DRPNN %
StarBrightness	0.5475	0.5763^*R*^	0.5873^*D*^^*R*^	-7.27%	-1.91%
Sunspot	3.7104^*D*^^*E*^	2.7781^*E*^	2.2596	39.1%	18.66%
EUR/USD	0.0078^*D*^^*E*^	0.007^*E*^	0.0057	26.92%	18.57%
Mackey–Glass	0.0185^*D*^^*E*^	0.0131	0.0121	34.59%	7.63%
**Average of improvement %**				23.34%	10.74%

These are the de-normalized results for 30 simulations.

^*E*^, means the RPNN-EF has significant performance using t-test;

^*D*^, means the DRPNN has significant performance using t-test;

^*R*^, means the RPNN has significant performance using t-test.

**Table 4:** Normalized Mean Squared Error (NMSE) improvement of RPNN-EF to RPNN and DRPNN.

	RPNN	DRPNN	RPNN-EF	Improvement to RPNN %	Improvement to DRPNN %
StarBrightness	0.0037	0.0041^*R*^	0.0043^*D*^^*R*^	-16.22%	-4.88%
Sunspot	0.0028^*D*^^*E*^	0.0016^*E*^	0.0011	60.71%	31.25%
EUR/USD	0.1072^*D*^^*E*^	0.0866^*E*^	0.0575	46.36%	33.6%
Mackey–Glass	0.0078^*D*^^*E*^	0.0034	0.0034	56.41%	≈ 0%
**Average of improvement %**				36.82%	14.99%

These are the de-normalized results for 30 simulations.

^*E*^, means the RPNN-EF has significant performance using t-test;

^*D*^, means the DRPNN has significant performance using t-test;

^*R*^, means the RPNN has significant performance using t-test.

**Table 5:** Mean Absolute Error (MAE) improvement of RPNN-EF to RPNN and DRPNN.

	RPNN	DRPNN	RPNN-EF	Improvement to RPNN %	Improvement to DRPNN %
StarBrightness	0.4450	0.4652^*R*^	0.4742^*D*^^*R*^	-6.56%	-1.93%
Sunspot	3.0029^*D*^^*E*^	2.2552^*E*^	1.8207	39.37%	19.27%
EUR/USD	0.0065^*D*^^*E*^	0.0057^*E*^	0.0048	26.15%	15.79%
Mackey–Glass	0.0142^*D*^^*E*^	0.0105	0.0097	31.69%	7.62%
**Average of improvement %**				22.66%	10.19%

These are the de-normalized results for 30 simulations.

^*E*^, means the RPNN-EF has significant performance using t-test;

^*D*^, means the DRPNN has significant performance using t-test;

^*R*^, means the RPNN has significant performance using t-test.

**Table 6:** Signal to Noise Ratio (SNR) improvement of RPNN-EF to RPNN and DRPNN.

	RPNN	DRPNN	RPNN-EF	Improvement to RPNN %	Improvement to DRPNN %
StarBrightness	35.8666	35.4226^*R*^	35.2549^*D*^^*R*^	-1.71%	-0.47%
Sunspot	37.7086^*D*^^*E*^	40.222^*E*^	42.0337	11.47%	4.5%
EUR/USD	39.6928^*D*^^*E*^	40.6037^*E*^	42.3944	6.81%	4.41%
Mackey–Glass	37.4584^*D*^^*E*^	40.0711^*E*^	41.44	10.63%	3.42%
**Average of improvement %**				6.8%	2.97%

These are the de-normalized results for 30 simulations.

^*E*^, means the RPNN-EF has significant performance using t-test;

^*D*^, means the DRPNN has significant performance using t-test;

^*R*^, means the RPNN has significant performance using t-test.

As seen from the four metrics results, the forecasting performance of the feedforward RPNN network is significantly better than the two recurrent networks for one-step ahead forecasting on the short time series (StarBrightness) (i.e., the symbol ^*R*^ is inside the cells that relate to DRPNN and RPNN-EF for StarBrightness series). Such results were found for one-step ahead forecasting with short time series in [60, 61]. The two recurrent networks DRPNN and RPNN-EF are significantly better than the feedforward RPNN network for one-step ahead forecasting on long time series (Sunspot) and for multi-step ahead forecasting on EUR/USD and Mackey–Glass time series. This means that the built memory during training in the recurrent networks for long series for one-step ahead forecasting and for multi-step ahead forecasting improves forecasting performance.

Results in Tables 3–6 show that average improvements in all metrics for RPNN-EF performance are more than twice in the case of RPNN than DRPNN. These findings prove that explicit treatment of dynamics helps to improve forecasting performance. Furthermore, using network error feedback as an input helps reduce the overall network error and improve forecasting performance.

As seen in average improvements for the four metrics, the highest average improvement for RPNN-EF is with NMSE metric while the lowest average improvement is with SNR metric. This is because the variables used in the equations for these metrics depend on the time series itself. For example, the variance variable used with NMSE and the maximum value used with SNR. Therefore, these variables increase or decrease metric values and average improvements.

**Fig 8:**
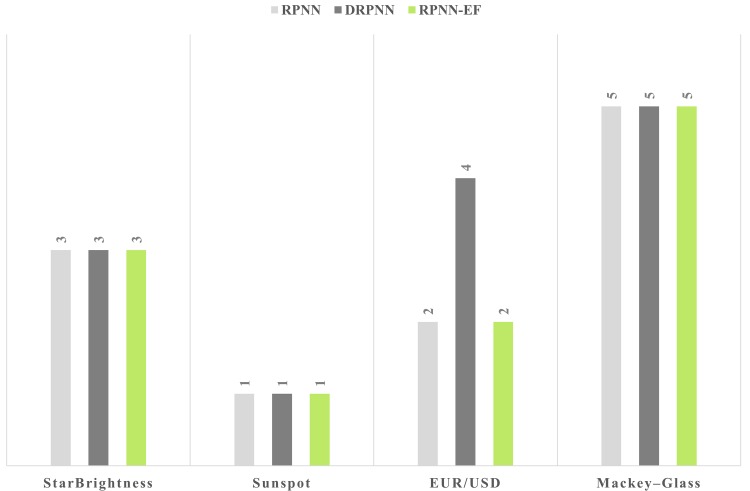
Network order for the best average simulations on the used time series.

Overall, RPNN-EF outperforms other RPNN based models, which is seen in average improvements across all metrics. This performance was achieved with network orders equal to or less than that of other RPNN based models as shown in Fig 8.

### Best Single Simulation Results

**Fig 9:**
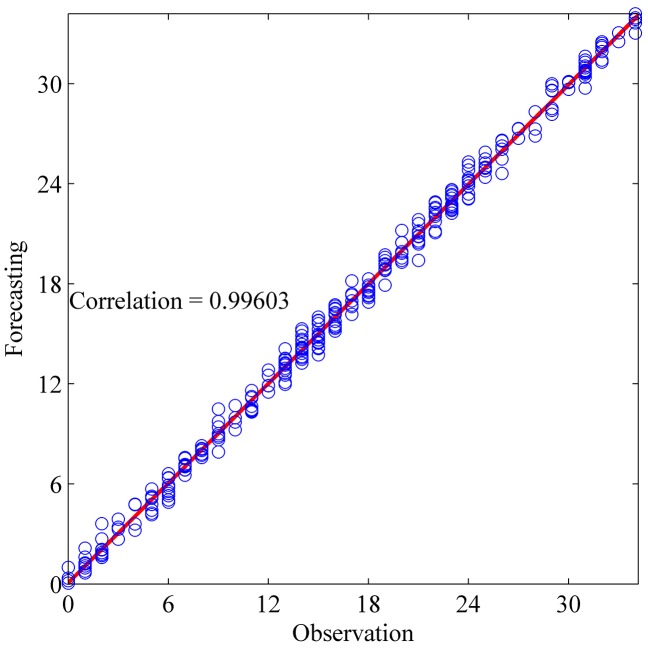
Correlation function between forecast and observed values for StarBrightness time series based on the best RPNN-EF simulation.

**Fig 10:**
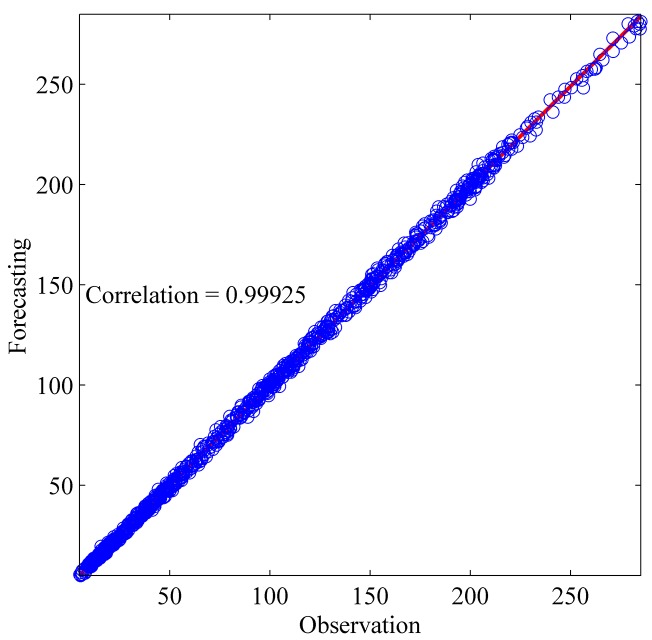
Correlation function between forecast and observed values for Sunspot time series based on the best RPNN-EF simulation.

**Fig 11:**
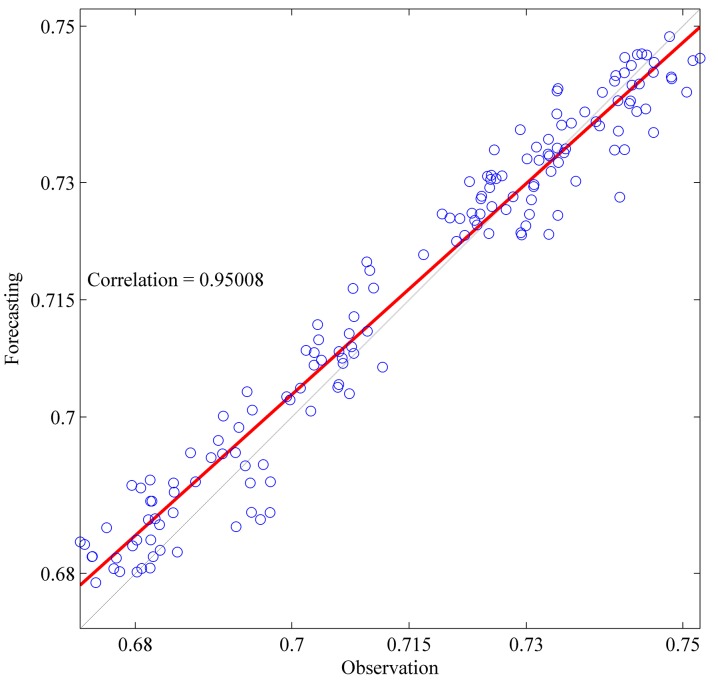
Correlation function between forecast and observed values for EUR/USD time series based on the best RPNN-EF simulation.

**Fig 12:**
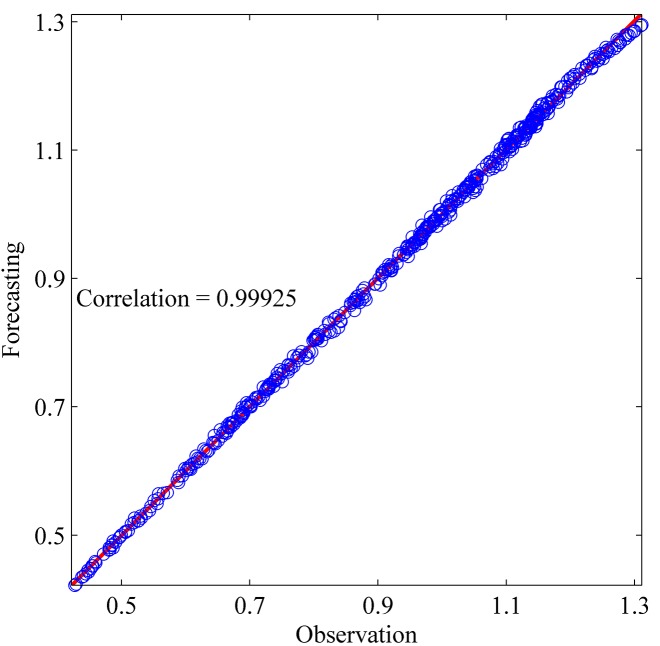
Correlation function between forecast and observed values for Mackey–Glass time series based on the best RPNN-EF simulation.

This section shows the results for the best single simulation achieved for each network model. As shown in Table 7, RPNN-EF has the smallest RMSE and NMSE values for all time series except for StarBrightness. Its performance for the StarBrightness time series is still acceptable. The forecast values were plotted with respect to observed values, as shown in Figs 9–12, which show a very strong relationship between forecasted and observed values for the Sunspot and Mackey-Glass time series. This is because the NMSE for these time series is very small when compared to other time series.

**Table 7:** Best Single Simulation Results.

Model	Order	RMSE	NMSE
StarBrightness			
RPNN-EF	3	0.5632	0.004
DRPNN	3	0.5468	0.0037
**RPNN**	3	**0.5372**	**0.0036**
SunspotNumbers			
RPNN	3	2.1594	0.001
DRPNN	3	1.9542	0.0008
**RPNN-EF**	3	**1.9088**	**0.0007**
EUR/USD			
RPNN	2	0.0072	0.0908
DRPNN	4	0.0068	0.0808
**RPNN-EF**	2	**0.0052**	**0.0477**
Mackey–Glass			
RPNN	4	0.0115	0.0026
DRPNN	5	0.0105	0.0022
**RPNN-EF**	5	**0.0062**	**0.0008**

These are the de-normalized results.

The best forecasting for RPNN-EF using out-of-sample data are shown in Figs 13–16. These figures indicate that the RPNN-EF model can follow the dynamic behaviour of the time series. The histogram for the forecasting errors of the best simulations for the time series are shown in Figs 17–20.

**Fig 13:**
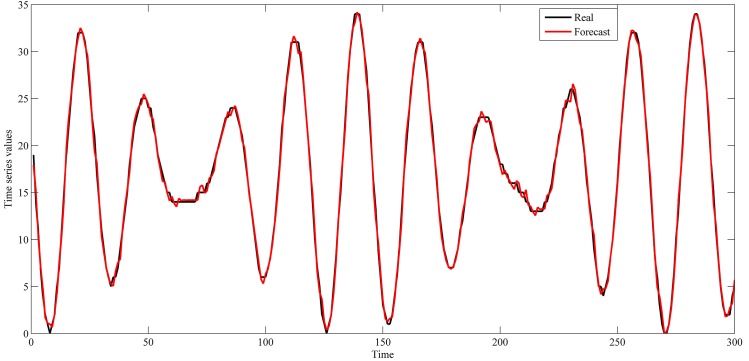
Out-of-sample forecasting for StarBrightness time series based on the best RPNN-EF simulation.

**Fig 14:**
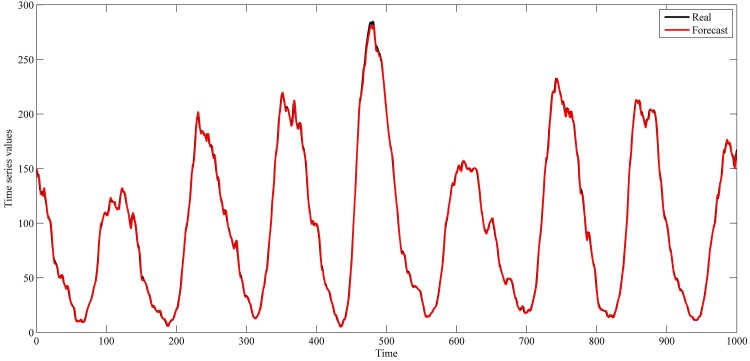
Out-of-sample forecasting for Sunspot time series based on the best RPNN-EF simulation.

**Fig 15:**
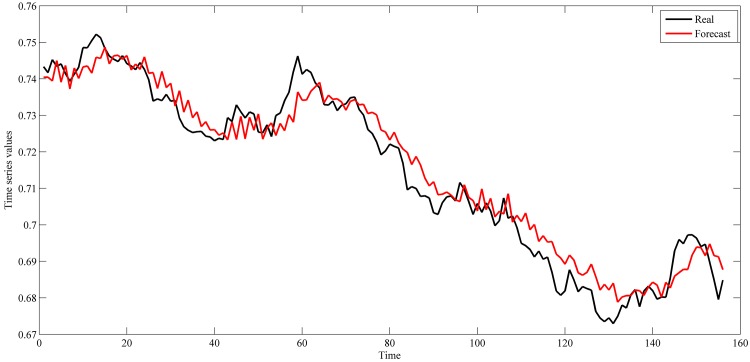
Out-of-sample forecasting for EUR/USD time series based on the best RPNN-EF simulation.

**Fig 16:**
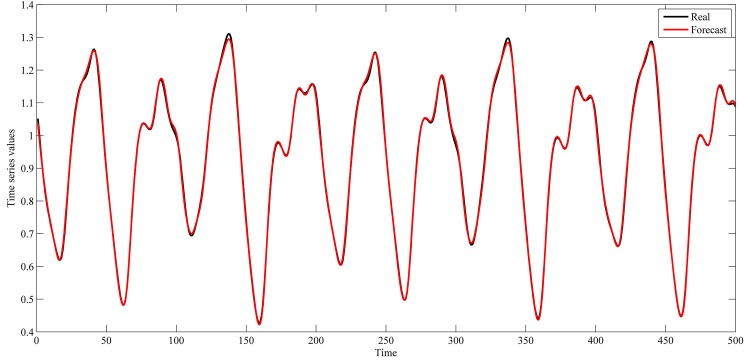
Out-of-sample forecasting for Mackey–Glass time series based on the best RPNN-EF simulation.

**Fig 17:**
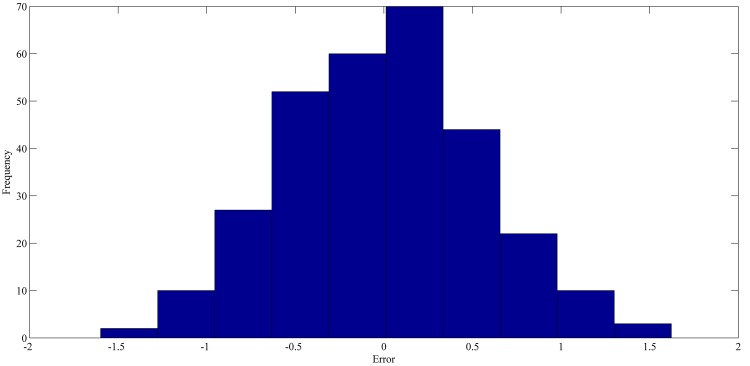
The histogram of the forecasting error for StarBrightness time series based on the best RPNN-EF simulation.

**Fig 18:**
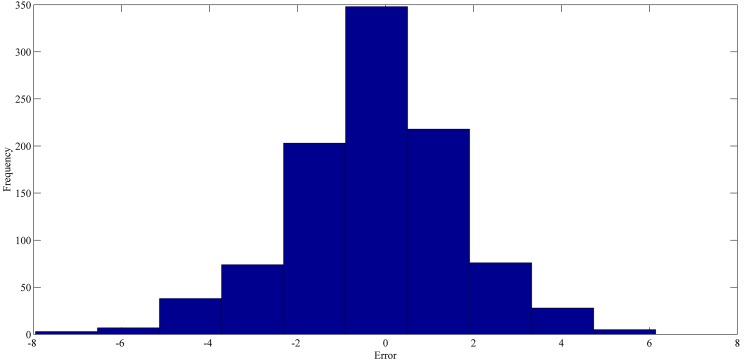
The histogram of the forecasting error for Sunspot time series based on the best RPNN-EF simulation.

**Fig 19:**
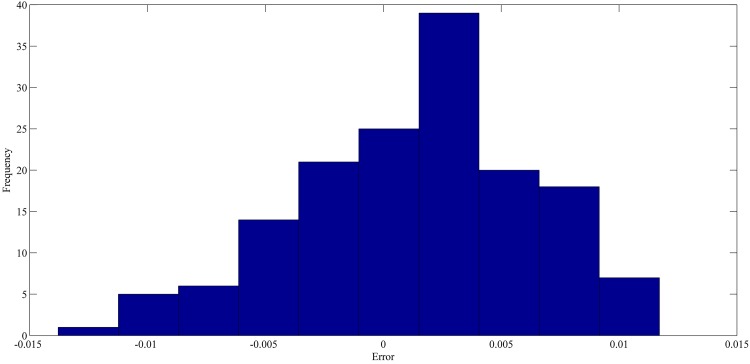
The histogram of the forecasting error for EUR/USD time series based on the best RPNN-EF simulation.

**Fig 20:**
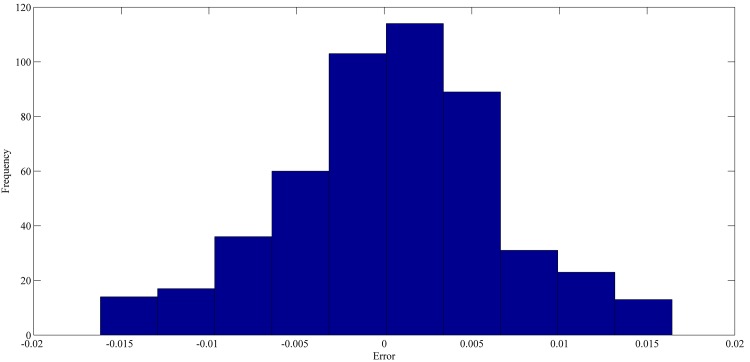
The histogram of the forecasting error for Mackey–Glass time series based on the best RPNN-EF simulation.

As seen in Figs 17–20, all histograms indicate that error distribution closely resembles a symmetric Gaussian distribution. Most of the errors are close to zero. This means the RPNN-EF is able to extract information from a time series.

### Comparison of the Performance of Various Existing Models

In this section, we compare our results with other models in the literature. Based on our search results, this study did not find studies that use the same normalization range as the present study. For a fair comparison with recent studies, this study compared the de-normalized results for the RPNN-EF model with de-normalized published results in the literature or with studies that did not use any normalization method.

Tables 8 and 9 show the comparison results for generalization capabilities using different methods for the Sunspot and Mackey-Glass time series, respectively. Generalization capabilities were measured by applying each model to forecast out-of-sample data. As was observed, RPNN-EF alone outperforms many hybrid methods. Therefore, hybridizing RPNN-EF with other models could produce higher forecasting accuracy.

**Table 8:** Comparison of the performance of various existing models on Sunspot series.

Model	RMSE	NMSE
FNN [42]	6.4905	0.0174
ART-FNN [42]	6.2204	0.0160
Modified-ART-FNN [42]	5.7173	0.0135
RPNN (used in this work)	2.1594	0.001
DRPNN (used in this work)	1.9542	0.0008
**RPNN-EF (proposed)**	**1.9088**	**0.0007**
Brain emotional learning-based RFS [43]	-	0.000664

ART, adaBoost.regression and threshold; FNN, fuzzy neural networks; DRPNN, dynamic ridge polynomial neural network; RFS, recurrent fuzzy system; RPNN, ridge polynomial neural network; RPNN-EF, ridge polynomial neural network with error feedback.

**Table 9:** Comparison of the performance of various existing models on Mackey–Glass series.

Model	RMSE
Fuzzy modeling method with SVD [49]	0.0894
Gustafson-Kessel fuzzy clustering method + KFA with SVD [50]	0.0748
Orthogonal function neural network + recursive KFA based on SVD [51]	0.05099
Adaptive fuzzy inference system with local research [52]	0.045465
Beta basis function neural networks + DE algorithm [53]	0.030
Backpropagation Network Optimized by Hybrid K-means-Greedy [54]	0.015
Multilayer feedforward neural network [55]	0.0155
Modified DE and the radial basis function [56]	0.013
RPNN (used in this work)	0.0115
DRPNN (used in this work)	0.0105
Functional-link-based neural fuzzy network-cultural cooperative PSO [57]	0.008424
WNN with the clustering based initialization approach [58]	0.0078
Flexible Beta Basis Function Neural Tree [59]	0.0068
MLMVN-QR decomposition [55]	0.0065
**RPNN-EF (proposed)**	**0.0062**
MLMVN [55]	0.0056

DE, differential evolution; DRPNN, dynamic ridge polynomial neural network; GD, gradient descent; KFA, kalman filtering algorithm; MLMVN, multilayer neural network with the multi-valued neurons; PSO, particle swarm optimization; RPNN, ridge polynomial neural network; RPNN-EF, ridge polynomial neural network with error feedback; SVD, singular value decomposition; WNN, wavelet neural network

### Learning Curve Results

**Fig 21:**
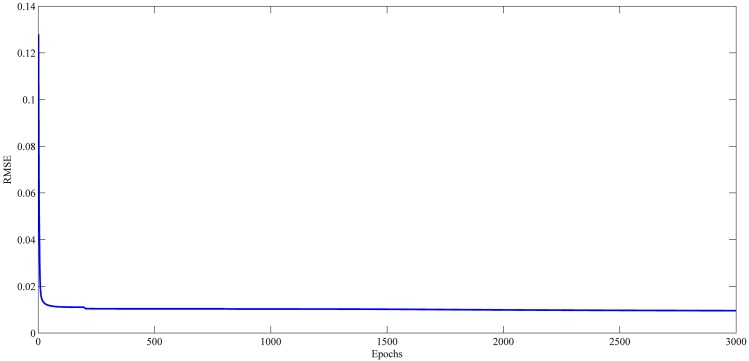
Learning curves for StarBrightness time series forecasting based on the best RPNN-EF simulation.

**Fig 22:**
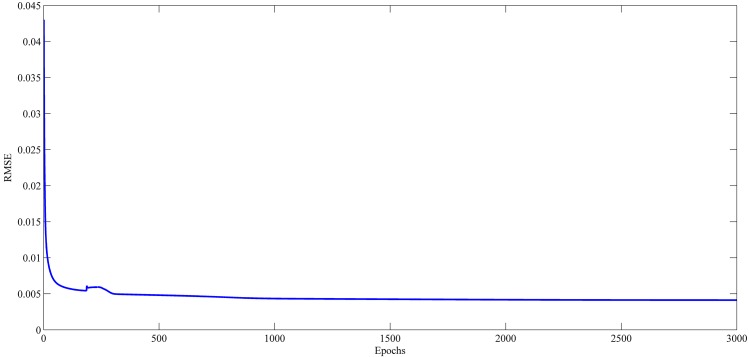
Learning curves for Sunspot time series forecasting based on the best RPNN-EF simulation.

**Fig 23:**
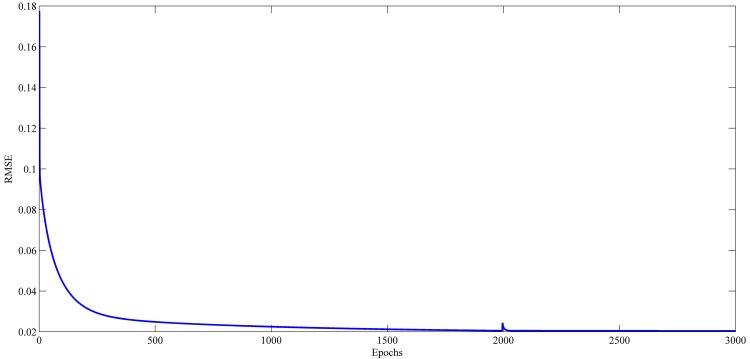
Learning curves for Daily Euro/Dollar (EUR/USD) exchange rate time series forecasting based on the best RPNN-EF simulation.

**Fig 24:**
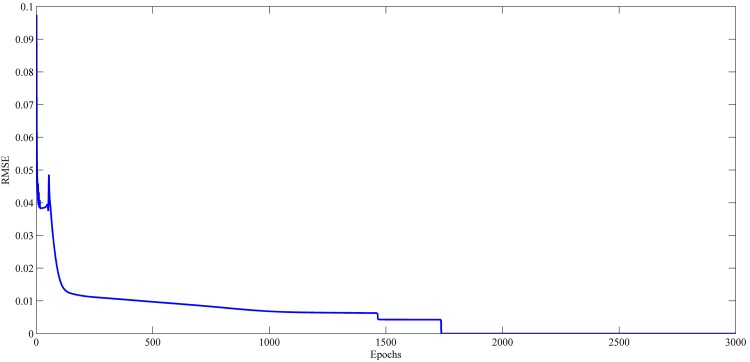
Learning curves for Mackey–Glass time series forecasting based on the best RPNN-EF simulation.

Figs 21–24 show the evolution of RMSE during the learning of RPNN-EF with the used time series. RPNN-EF has the ability to quickly converge. Note that the RMSE values in Figs 21–24 are normalized values.

For all time series, the learning curves for RPNN-EF are remarkably stable and RMSE continuously reduced every time Pi-Sigma block is added to the network. Each spike shown in Figs 21–24 comes from the introduction of a new Pi-Sigma block to RPNN-EF except for a spike in the Mackey-Glass time series at epoch 54, which is due to an increase in RMSE, that rarely occurs with the sufficient condition due to the small values of the input signal as found in [62].

## Conclusions and future work

In this study, a new approach called Ridge Polynomial Neural Network with Error Feedback (RPNN-EF) for time series forecasting was proposed. The goal of this study is to contribute a new approach for time series forecasting that take advantage of higher order terms, recurrence, and error feedback. This study demonstrated the effectiveness of the proposed model by testing it on four time series for one-step and multi-step ahead forecasting. This study compared RPNN-EF with the feedforward Ridge Polynomial Neural Network (RPNN) and the Dynamic Ridge Polynomial Neural Network (DRPNN). The results of the study are summarized as follows:

- Recurrent networks are more suitable than feedforward networks for multi-step ahead forecasting.
- For one-step ahead forecasting with long training data, recurrent networks are better than feedforward networks because the dynamics of the time series captured and saved in the recurrent network’s memory. For short training data, the dynamics of the time series are not captured well might due to the short length of the training samples.
- Using network error feedback as an input helps reduce the overall network error more often than network output feedback, thus improving forecasting performance. Showing a network the difference between the desired output and its real output, which is known as error, during training helps enhance the overall forecasting performance for the network.
- Although RPNN-EF has the highest average performance, it uses network orders equal to or smaller than other RPNN models.
- Sufficient conditions to ensure RPNN-EF stability helps RPNN-EF to become stable in most cases.

With regards to model development, the following can be considered for future investigation:

- Applying the proposed model with more time series for one-step and multi-step ahead forecasting with different lengths to prove forecasting performance.
- This study focuses on univariate time series, which is data from a single time series. The ever more global nature of some series such as the world financial markets necessitates the inclusion of more global knowledge into neural network design. Multivariate series can look at the interdependence between several time series. Therefore, the use of multivariate series would be advantageous, since some market depends on other global markets and the inclusion of these series will potentially improve neural network forecasting performance.
- Like other ridge polynomial based models, the main difficulty of using RPNN-EF is finding suitable values for its parameters. With respect to this deficiency, it might be worthwhile to consider how evolutionary and swarm intelligence techniques can be used to automatically generating suitable parameters for the network. Furthermore, these techniques can be used to optimize network weights.
- To increase the reliability of forecasting, an ensemble system that uses RPNN-EF with another techniques can be proposed.
- The use of error feedback recurrence with other neural network models and an evaluation of their forecasting performance.
